# Oral health among children participating in an extended home visiting programme—a case‒control study

**DOI:** 10.1007/s40368-025-01009-6

**Published:** 2025-02-19

**Authors:** I. Brännemo, J. Norman, T. Kvist, L. Lindberg, G. Tsilingaridis

**Affiliations:** 1 Division of Pediatric Dentistry, Department of Dental Medicine, Karolinska Institutet, Stockholm, Sweden; 2 Center for Pediatric Oral Health Research, Stockholm, Sweden; 3 UWA Dental School, The University of Western Australia, Nedlands, WA Australia; 4 Department of Global Public Health, K9, Karolinska Institutet , Stockholm, Sweden; 5 Center of Epidemiology and Community Medicine, Stockholm, Sweden

**Keywords:** Child health services, Early childhood caries, Parental education, Preventive dentistry, Social services, Socioeconomic disadvantage

## Abstract

**Purpose:**

To evaluate whether an extended home visiting programme by child health nurses and parent counsellors can prevent caries and improve oral health habits in children from low socioeconomic backgrounds.

**Methods:**

All families in Sweden are offered one home visit when the child is two weeks old. The extended home visiting programme included five extra home visits between two and fifteen months of age where every visit had a specific theme (child safety, feeding, attachment/interaction, parenthood, social network, and self-care). Toothbrush and toothpaste, together with oral health information, were given around eight months of age. Clinical examinations and oral health habit questionnaires were conducted at 12, 24, and 36 months of age. The International Caries Detection and Assessment System was used to assess caries, and the results were compared to those of an age-matched control group following the standard child health care programme.

**Results:**

Significantly (*p* < 0.05) more children had caries at 36 months of age in the intervention group (32.8%) than in the control group (10.1%). The intervention group had significantly (*p* < 0.05) more plaque and a greater frequency of sweet snacks and drinks. In the intervention group, significantly (*p* < 0.05) more parents were under the age of 25 and foreign-born. Significantly (*p* < 0.001) more foreign-born parents were born in high-income countries in the control group compared to the intervention group.

**Conclusion:**

The extended home visiting programme did not improve the prevalence of caries or oral health habits in this cohort. However, since the groups differed in socioeconomic factors, the results should be interpreted with caution.

## Introduction

Early childhood caries (ECC) is a common chronic disease particularly affecting children in areas with low socioeconomic status (Bernabe et al. 2020; Tinanoff et al. 2019). Pain and infection, as a result of caries, can affect food intake and sleeping habits, resulting in decreased growth and a negative effect on a child’s quality of life (Acs et al. 1999; Tinanoff et al. 2019). ECC also increases the risk of caries later in life (Fontana 2015) and future dental anxiety (Raadal et al. 2002), highlighting the importance of keeping young children cavity free.

The aetiology of caries is multifactorial and involves clinical, social, and behavioural risk factors. Clinical risk factors include previous caries experience, dental biofilm, cariogenic microflora, enamel defects, genetic susceptibility and saliva quality/quantity (Tinanoff et al. 2019; World Health Organization, 2019). Moreover, social and behavioural risk factors include frequent intake of sugars (fermentable carbohydrates), low health literacy, poverty, caries among caregivers, maternal smoking and obesity, nutritional status in mothers and babies, not using fluoridated toothpaste, and frequent/nocturnal feeding beyond 12 months of age (Julihn et al. 2018; Tinanoff et al. 2019; World Health Organization, 2019). Low socioeconomic status, along with immigrant background, ethnic minority status, and low maternal education levels, is associated with increased caries prevalence and risk (Kim Seow, 2012), with a more pronounced association in young children (Kramer et al. 2018). Additionally, Swedish studies have shown that children with foreign-born parents have a higher caries prevalence (Granlund et al. 2022), a lower frequency of toothbrushing and more frequent snacking habits (Stecksén-Blicks et al. 2008).

Caregivers are responsible for their child’s diet and oral health habits. Knowledge of oral health and self-efficacy have been highlighted as facilitators of supervised toothbrushing, whereas a lack of knowledge and difficulties in managing child behaviour serve as barriers (Aliakbari et al. 2021). Caries risk is also greater in children whose parents rate their ability to control toothbrushing and sugar intake as low (Pine et al. 2004). This indicates the need to communicate oral health information to parents and also strengthen parents’ self-efficacy and provide tools to manage child behaviour. Home visits have been suggested as early interventions for preventing ECC (Tinanoff et al. 2019), with promising results in Australia (Plonka et al. 2013), the United Kingdom (Kowash 2000) and Sweden (Brännemo et al. 2021).

Since early prevention is essential, collaboration with other professionals meeting children early in life is an important arena of collaboration (Swedish National Board of Social Affairs and Health, 2022). The Child Health Services (CHS) in Sweden reaches almost 99% of all children, is free of charge and offered to all families from birth until the age of six years (Burström et al. 2017; Reuter 2018). Prenatal and/or postnatal dental programmes, delivered by nondental professionals to mothers, have shown promising results in decreasing ECC in a recent review (George et al. 2019).

The extended home visiting programme is an ongoing intervention for early health promotion and prevention in Stockholm, Sweden. As a part of the standard CHS programme, all parents in Sweden are offered one home visit by a CHS nurse at the child’s age of two weeks. The extended home visiting programme is a collaboration between the CHS and the social services, targeting first-time parents in socioeconomically vulnerable areas, with five additional home visits when the child is between two and fifteen months of age. The home visits are performed by a CHS nurse and a parent counsellor from social services aiming to improve child health. The included themes are “child safety, child feeding, child attachment/interaction, parenthood, social network, and selfcare” (Burström et al. 2017), with an emphasis on creating a dialogue with the family. All families receiving the extended home visiting programme also attend the standard CHS programme with regular visits to the CHS clinic (Burström et al. 2017; Melbom et al. 2018). During the fourth home visit, when the child is around eight months of age, parents are given a toothbrush and a toothpaste (1000 ppm), together with recommendations on the importance of toothbrushing with fluoride toothpaste when the first tooth erupts. Furthermore, the discussion covers routines and strategies for guiding a child, using toothbrushing as an example, along with advice on maintaining a healthy diet (Melbom et al. 2018). The aim of this study was to examine whether an extended home visiting programme, delivered by CHS nurses in collaboration with parent counsellors from social services, prevents caries and positively affects oral health habits among children living in areas with low socioeconomic status. Our hypothesis is that children participating in the extended home visiting programme will have less caries and better oral health habits at 36 months of age compared to children receiving the standard CHS programme.

## Materials and methods

### Participants

Children and parents (*n* = 253) at nine CHS clinics in Stockholm County, previously included in the overall evaluation of the extended home visiting programme (Mekhail et al., 2024), were invited to dental examinations. Five clinics offering the extended home visiting programme served as the intervention group, and four clinics following the standard CHS programme served as the control group. The clinics in the intervention group were randomised from clinics already using the home visiting programme, whereas clinics with similar populations and social structures, not using the home visiting programme, were randomized as the control group. Families in the intervention group were excluded if they received less than two home visits (The Public Health Agency of Sweden, 2022). The project areas are marked by lower socioeconomic status, with residents having lower incomes, lower education levels, higher unemployment, more sick days, and a higher proportion of foreign-born individuals compared to the average population in Stockholm County (Region Stockholm Centre for Epidemiology and Community Medicine 2024).

### Data collection

Oral health data were collected through a clinical examination and a questionnaire at the ages of 12, 24, and 36 months. Owing to the nature of the study, the examiners were not blinded. Knee-to-knee position or examination in the dental chair was used depending on age and child cooperation. The teeth surfaces were dried with cotton rolls and examined with a mouth mirror. If needed, a toothbrush was used to remove plaque, and the presence of plaque and gingivitis were recorded (yes/no). Caries lesions were registered on a surface level ranging from zero to six following the International Caries Detection and Assessment System (ICDAS). A caries-free surface was coded as zero, initial caries only visible when dry as one, initial caries visible when wet as two, enamel breakdown with no visible dentin as three, cavity in enamel with a shadow in dentin as four, a cavity involving dentin as five and a cavity involving dentin extending over more than half the surface as six (Pitts and Ekstrand, 2013). In conjunction with the clinical examination, the parents answered a questionnaire adapted from earlier Swedish studies on known risk factors (Brännemo et al. 2021; Julihn et al. 2006). Oral hygiene habits, such as toothbrushing and snacking, were measured as never/up to once a week/2–3 times per week/4–6 times per week/every day. Examples of other questions included snacking between meals (yes/no) and nocturnal drinks or meals (yes/no). The questionnaire also included a question on previous dental trauma (yes/no). In addition, parents were interviewed at 0–2 months (preintervention) and 18 months (postintervention) to collect background data to evaluate the effects of the programme as a whole (Mekhail et al., 2024). These data included parental age, education length, income source, birth country and interview language together with self-efficacy score (parental self-efficacy) (Ulfsdotter et al. 2014) and health literacy score (HLS-EU-Q16) (Mekhail et al. 2022).

### Calibration

Four examiners (three paediatric dentists and one dental hygienist with experience in paediatric dental care) were calibrated in caries diagnostics using ICDAS. The calibration included a 90-min e-learning programme (Topping et al. 2008), a half-day lecture programme and calibration exercises. Furthermore, an interexaminer calibration exercise was performed with 25 pictures of single teeth affected with caries, followed by a joint clinical examination of two patients. The picture-based portion of the calibration exercise was repeated after one month. The intraexaminer reliability was calculated as follows: mean *k*_*w*_ = 0.92 (range: 0.85–0.98). Interexaminer reliability was calculated by comparing the examiners’ results with those of a standard previously set by experienced examiners (Anderson et al. 2016). At the first occasion, the mean interexaminer reliability was *k*_*w*_ = 0.94 (range: 0.87–1.00), followed by *k*_*w*_ = 0.95 (range: 0.92–0.98). The total mean interexaminer reliability was 0.95 (range: 0.87–1.00).

### Statistical analysis

SPSS Statistics version 29.0.1.0 for Windows was used to analyse the data. Descriptive analysis consisted of mean number, standard deviation, and frequency, including the mean number of cavitated teeth/surfaces and the proportion of patients in the intervention group and control group. For variables with a non-normal distribution, the median and range are presented. The Chi-square test or Fisher’s exact test was used to evaluate the difference between the proportions of characteristics of participants and differences in caries progression between examinations and between groups. Yate’s continuity correction was used for 2 × 2 tables. For continuous variables, the Mann‒Whitney U test or t-test was used to assess differences in the mean number of decayed surfaces between groups and between examinations. A generalized linear mixed-effects model for repeated measures was used to measure the effect of the intervention by calculating the change in the ICDAS score between baseline and the two follow-ups. The significance level was set at *p* < 0.05. Qualitative and sociodemographic data differed because one or two parents were interviewed, and the data were analysed in the same way as by the researchers evaluating the programme as a whole (The Public Health Agency of Sweden, 2022). For the variables presenting “parent”, the mothers’ answer was chosen if available, and the fathers’ answer was chosen if the mother had not answered. In cases with two mothers or fathers, the person marked “one” in the data was chosen. The family mean was calculated for education length, years living in Sweden if foreign-born, health literacy and self-efficacy. Parental age was divided into categories < 25, 25–34 and > 34 years on the basis of a previous study on caries and maternal age (Julihn et al. 2018). The income level of parents’ birth country was categorized according to the World Bank, and lower-middle income and higher-middle income were comprised to “middle income” (World Bank. World Developement Indicators, 2023). The income source was composed by combining all forms of mixed income into one and all types of social aid into one. Toothbrushing morning and night, the use of fluoride and bleeding when brushing were comprised into daily/not daily.

## Results

In total, 181 children were examined at 12 months of age (intervention: 88, control: 93), followed by 147 children at 24 months of age (intervention: 70, control 77) and 133 children at 36 months of age (intervention: 64, control: 69). The reasons for not being examined were cancelation, no show, declining participation, or unknown reasons. There were no significant differences in baseline characteristics between the groups regarding sex, age at first exam, years in Sweden for foreign-born parents, parental income source, education length or interview language (Table 1). Significantly more parents in the intervention group were under 25 years of age at the birth of the child (*p* = 0.014). There was a greater proportion of foreign-born parents in the intervention group (*p* = 0.001). Furthermore, among the foreign-born parents, there were significantly more parents from low-income and middle-income countries in the intervention group and from high-income countries in the control group (*p* =  < 0.001) (Table 1).

**Table 1:** Descriptive data of the included children and parents before the start of the intervention (0–2 months of age)

	Intervention		Control		
	*%*	*n*	*%*	*n*	*p*
Children					
Girls	50.0	44	46.2	43	0.721
Boys	50.0	44	53.8	50	
Age child, first exam (months)^b^					
Median (min–max)	12.0 (10.0–16.0)		12.0 (10.0–14.0)		0.136
Age parent, at birth (years)					
< 25	**14.6**	13	**5.2**	5	**0.014**
25–34	**59.6**	53	**78.4**	76	
> 34	25.8	23	16.5	16	
Parent born in Sweden					
Yes	32.6	29	57.4	60	**0.001**
No	67.4	60	42.6	40	
Years in Sweden (family mean)					
Median (min–max)	6.0 (0.5–28)		5.5 (0.75–33)		0.721
Income level parents birth country					
Not classified	1.1	1	0	0	** < 0.001**
Low income	**13.5**	12	**1.1**	1	
Middle income	**39.3**	35	**24.5**	23	
High income	**46.1**	41	**74.5**	70	
Income source					
No income	0.0	0	1.0	1	0.485
Social aid	22.2	20	32.3	31	
Partners income	12.2	11	8.3	8	
Student loan	4.4	4	3.1	3	
Mixed income	12.2	11	14.6	14	
Employment	48.9	44	40.6	39	
Education length (family mean)					
Mean (SD)	14.8 (2.9)		15.5 (2.7)		0.105
Interview language					
Swedish	70.0	63	79.4	77	0.302
English	14.4	13	11.3	11	
Other (interpreter)	15.6	14	9.3	9	

Bold indicates statistical difference (*p* < 0.05)

### Caries

In total, 21.2% of the children showed any sign of caries lesions, including both cavitated and noncavitated lesions (ICDAS scores of 1–6), at 36 months of age. Cavitated lesions were observed in 6.8% of the children (ICDAS 3–6). Significantly more children had any sign of caries (ICDAS scores of 1–6) at 36 months of age in the intervention group (32.8%) than in the control group (10.3%) (*p* = 0.003). Noncavitated caries lesions alone (ICDAS scores of 1–2) were also more common in the intervention group (28.1%) than in the control group (7.2%) (*p* = 0.003). However, there was no significant difference in cavitated caries lesions between the groups (Table 2). A generalized mixed model revealed no significant effect of the intervention on differences in caries progression (Δ ICDAS score) between the groups (*p* = 0.120). The result remained nonsignificant when controlling for parental age, parents not born in Sweden, income level of foreign-born parents’ birth country and health literacy before the intervention started. However, when both groups were analysed together, caries was significantly more common among children with parents under the age of 25 (*p* = 0.009), parents born outside of Sweden (*p* = 0.002) and parents born in middle-income countries (*p* = 0.010).

**Table 2:** Differences between the intervention group and controls regarding the prevalence of caries, plaque, gingivitis, and trauma at 12, 24 and 36 months of age

	Age 12 months			Age 24 months			Age 36 months		
	Intervention	Control		Intervention	Control		Intervention	Control	
	% (*n*)	% (*n*)	*p*	% (*n*)	% (*n*)	*p*	% (*n*)	% (*n*)	*p*
Deft^*^ mean (SD)				0.17(0.96)	0(0)	0.067	0.31(1.30)	0.07(0.36)	0.247
ICDAS^**^									
Total caries (1–6)	2.3(2)	1.1(1)	0.615	24.3(17)	5.2(4)	**0.002**	32.8(21)	10.3(7)	**0.003**
Noncavitated caries (1–2)	2.3(2)	1.1(1)	0.615	24.3(17)	5.2(4)	**0.002**	28.1(18)	7.2(5)	**0.003**
Cavitated caries (3–6)				4.3(3)		0.106	9.4(6)	4.4(3)	0.314
Plaque	14.8(13)	9.8(9)	0.427	24.3(17)	18.2(14)	0.482	32.8(21)	16.2(11)	**0.043**
Gingivitis	2.3(2)	2.2(2)	1.000	2.9(2)		0.225	1.6(1)	1.5(1)	1.000
Trauma	23.9(21)	29.0(27)	0.536	41.2(28)	32.9(25)	0.392	22.2(14)	15.9(11)	0.486

^*^Decayed, extracted, filled, teeth

^**^International caries detection and assessment system

### Oral health

Plaque was found in 32.8% of the children in the intervention group compared to 16.2% in the control group at 36 months of age (*p* = 0.043). There were no significant differences at 12 months of age (*p* = 0.427) or 24 months of age (*p* = 0.482). Similarly, there were no significant differences in dental trauma or gingivitis at any age (Table 2).

### Oral health habits

A significantly greater proportion of children in the intervention group consumed sweet snacks between meals at 12 months of age (*p* = 0.025) and drank sweet drinks between meals at 24 months of age (*p* = 0.016). However, there were no significant differences at other ages or in nocturnal intakes/breastfeeding at any age. The majority of the parents in both groups brushed their child’s teeth daily, with no significant difference at any age. At 12 months of age, 65.9% of the parents in the intervention group and 79.3% of the parents in the control group brushed their child’s teeth daily (*p* = 0.063). The proportion of parents brushing their child's teeth daily increased in both groups at each examination (Table 3).

**Table 3:** Oral health habits at 12, 24 and 36 months of age

	Age 12 months			Age 24 months			Age 36 months		
	Intervention	Control		Intervention	Control		Intervention	Control	
	% (*n*)	% (*n*)	*p*	% (*n*)	% (*n*)	*p*	% (*n*)	% (*n*)	*p*
More than 5 intakes/day	51.1 (45)	57.0 (53)	0.522	47.1 (33)	37.3 (29)	0.320	17.5 (11)	24.6 (17)	0.427
Sweet snacks between meals	27.3 (24)	12.9 (12)	**0.025**	12.9 (9)	20.8 (16)	0.290	17.5 (11)	17.6 (12)	1.000
Sweet drinks between meals	22.1 (19)	11.8 (11)	0.102	35.7 (25)	16.9 (13)	**0.016**	14.3 (9)	14.7 (10)	1.000
Nocturnal intake	40.9 (36)	26.9 (25)	0.066	22.9 (16)	22.1 (17)	1.000	7.9 (5)	17.4 (12)	0.174
Nocturnal breastfeeding	33.0 (29)	46.2 (43)	0.094	7.1 (5)	10.4 (8)	0.688	3.2 (2)	1.4 (1)	0.606
Toothbrushing daily	65.9 (58)	79.3 (73)	0.063	85.7 (60)	96.1 (73)	0.057	90.5 (57)	97.1 (67)	0.220
Fluoride toothpaste daily	69.3 (61)	76.3 (71)	0.370	92.9 (65)	97.4 (75)	0.258	90.5 (57)	95.7 (66)	0.309

### Parental knowledge

At baseline, there was a significant difference in health literacy, with a median score of 51 in the intervention group (range: 16–64) and 52 in the control group (range: 37–64) (*p* = 0.045). There was no significant difference at follow-up, where the median was 50 (range: 36–64) in the intervention group and 50 (range: 40–64) in the control group. The median self-efficacy score was 72 (range: 48–80) at baseline in the intervention group and 71 (range: 28–80) in the control group. There was no significant difference in the mean self-efficacy score at any timepoint, and there was no significant correlation between health literacy and self-efficacy with caries prevalence, toothbrushing or sweet intakes.

## Discussion

In this study, the extended home visiting programme did not have a beneficial effect on caries or oral health habits in comparison to children following the standard CHS programme.

With all signs of caries included, a total of 21% had caries at three years of age, emphasizing the importance of measuring noncavitated caries lesions and the need for early prevention. These results are in line with another Swedish study using ICDAS, where 22–23% of the children in an area with low socioeconomic status had caries lesions (Anderson et al. 2016).

Toothbrushing reported by parents increased in both groups at each exam, but there was no significant difference between the groups. However, 44.1% of the parents in the intervention group did not brush their child’s teeth daily at one year of age even though they were given oral health information, toothpaste, and a toothbrush between six and eight months of age. In comparison, 14% did not brush daily at 18 months in a previous study on the same home visiting programme (Brännemo et al. 2021). Another study in similar areas in Stockholm reported that 47% did not brush their child’s teeth daily at 12 months of age (Anderson et al. 2016). However, self-reported toothbrushing might not necessarily mean good oral hygiene, and parents may overestimate the frequency of toothbrushing.

Parental self-efficacy and knowledge have been reported as facilitators of parental toothbrushing (Aliakbari et al. 2021). A prenatal programme on oral health delivered by midwives improved oral health knowledge (George et al. 2018). Despite the greater degree of oral health knowledge among mothers in the intervention group, a follow-up study revealed no differences in the prevalence of oral health, diet, or caries among their children (George et al. 2023). This suggests that knowledge alone is insufficient. Notably, both groups in the present study received information on oral hygiene and dietary habits at the time of the dental exams at 12, 24 and 36 months of age, which may have affected the results. A previous study on health literacy among parents participating in the current extended home visiting programme revealed no difference compared with parents not participating. However, among parents who needed an interpreter, health literacy was significantly improved in the intervention group (Mekhail et al. 2023). The present study revealed no correlation between health literacy and caries or oral health habits.

Collaboration with nondental professionals for caries prevention is recommended both globally (Peres et al. 2019; Tinanoff et al. 2019) and in Sweden (National Board of Social Affairs and Health, 2022). Previous studies in the United Kingdom and Australia have shown that home visiting programmes can prevent caries (Kowash 2000; Plonka et al. 2013). In contrast to the study evaluating the first group participating in the extended home visiting programme in Stockholm, where caries were more common in the control group (Brännemo et al. 2021), this study revealed a larger proportion of children with caries in the intervention group. Similarly, a recent study on an ECC preventive programme starting during pregnancy in another region in Sweden revealed no effect on caries prevention (Blomma et al., 2024). In contrast, a Swedish parental support programme between two and five years of age in a low socioeconomic area in Malmö showed lower caries prevalence. However, four percent of the three-year-olds in the study had already gone through caries treatment, suggesting the need for earlier invention (Wennhall et al. 2008).

The cost for the whole extended home visiting programme is approximately 4000 SEK per child (Mekhail 2022). However, there is no additional cost for the oral health promotion, which potentially could decrease the need for dental appointments at early age which would benefit both dental care and families. A cost-analysis for an extended caries-preventive programme performed by the dental care in similar areas in Stockholm was not shown to be cost-effective. (Anderson et al. 2019) Including oral health in the regular check-ups at CHS could also be a way for a cost-effective early collaboration in prevention.

More caries were found among children with foreign-born parents and parents born in middle-income countries in this study, which was also reported in a previous Swedish study (Granlund et al. 2022). The age of the mother was important for caries development in another study, with more caries among children with mothers under the age of 25 and over the age of 35 (Julihn et al. 2006). Similarly, this study revealed more caries lesions among children whose parents were under the age of 25 years than among those whose parents were over 35 years, but the difference was not significant. Moreover, more parents under the age of 25, foreign-born parents and parents from middle-income countries were included in the intervention group than in the control group in this study. These differences in socioeconomic factors, which were previously shown to be risk factors for dental caries, may have affected the results.

The differences in background factors between the groups are a limitation, possibly stemming from the selection of the CHS clinics. The selection was based on parameters other than oral health, and the prevalence of caries in the participating areas differed between the intervention group and the control group (Health and Medical Care Administration, 2023). The lack of blinding was a limitation, as it was not feasible due to the nature of the intervention. Additionally, the study was impacted by the COVID-19 pandemic, leading to some home visits being conducted digitally, at CHS clinics, or cancelled altogether. Families receiving less than two home visits were excluded, as some families might not have received information on oral health as planned. In some families, two parents were interviewed, and in others, only one parent was interviewed, indicating a possible difference when mean scores were used. Lastly, the short follow-up period and small sample size represent limitations that could affect the study’s ability to detect long-term effects or ensure generalizability and should be addressed in future research.

A strength with the present study, and the overall evaluation, is inclusion of all first-time parents in the study areas reflecting reality. Furthermore, there were no exclusion due to language barriers, which is common in areas with low socioeconomic status. An interpreter was booked free of charge upon request and consent forms were available in several languages. Another strength is the registration of both non-cavitated and cavitated caries lesions, depicting a more comprehensive caries prevalence aiding objectivity by being assessed through a standardised registration (ICDAS).

## Conclusion

To conclude and given the limitations of the present study it has been shown that compared with the standard CHS programme, the extended home visiting programme did not have a beneficial effect on caries development or oral health habits. Further studies are needed to evaluate the effects on oral health, as home visiting programmes are being implemented on a larger scale in Sweden. Since the comparison groups in this study differed in socioeconomic factors known to affect caries development, the results should be interpreted with caution, and these factors need to be taken into consideration in future research plans.

## Data Availability

Data that support the findings of this study are available from the corresponding author upon request.
